# Revisiting the taxonomy of *Fagopyrum caudatum* var. *grandiflorum* (Polygonaceae) using morphological and molecular data

**DOI:** 10.3897/phytokeys.276.196051

**Published:** 2026-06-18

**Authors:** Lu Tan, Ron Yan, Han-Mei Du, Ying Peng, Zi-Xin Fan, Qing-Hai Wang, Ana Valdés-Florido, Hong Chen, Sheng-Chun Li, An-Hu Wang

**Affiliations:** 1 Panxi Crop Improvement Key Laboratory of Sichuan Province, Xichang University, Xichang 615000, Sichuan, China Panxi Crop Improvement Key Laboratory of Sichuan Province, Xichang University Xichang China; 2 Triticeae Research Institute, Sichuan Agricultural University, Wenjiang 611130, Sichuan, China College of Agricultural Science, Xichang University Xichang China; 3 Department of Plant Biology and Ecology, University of Seville, Seville, Spain Department of Plant Biology and Ecology, University of Seville Seville Spain; 4 College of Agricultural Science, Xichang University, Xichang 615000, Sichuan, China Triticeae Research Institute, Sichuan Agricultural University Wenjiang China

**Keywords:** Chloroplast region, classification, ITS, leaf anatomy, palynology, phylogeny

## Abstract

*Fagopyrum caudatum* var. grandiflorum M.L.Zhou & Yu Tang is a perennial herb found in Gansu Province of China. In the present study, we combined morphological, palynological, anatomical and genetic data to clarify the taxonomic status of *F. caudatum* var. grandiflorum. Morphologically, in terms of flower size, achene size, leaf shape, and petiole characteristics, *F. caudatum* var. grandiflorum shows significant differences with *F. caudatum*. Its pollen grains also differ notably in size, colpi length and width, and lumina size compared to *F. caudatum*. Anatomically, the upper epidermis, lower epidermis, palisade tissue, spongy tissue, and leaf thickness of *F. caudatum* are greater than *F. caudatum* var. grandiflorum. Phylogenetic analysis based on ITS sequences indicates that *F. caudatum* var. grandiflorum has a closer genetic relationship to *F. callianthum* and *F. lineare* than to *F. caudatum*. Meanwhile, chloroplast regions analysis shows that *F. caudatum* has a closer genetic relationship with *F. macrocarpum*. Overall, the results from morphology, palynology, leaf anatomy, and phylogenetics support that the relationship between *F. caudatum* var. grandiflorum and *F. caudatum* should be interspecific rather than infraspecific. Considering morphological and geographic distribution, it is appropriate to recognize *F. caudatum* var. grandiflorum as a distinct species, renamed *Fagopyrum grandiflorum* (M.L.Zhou & Yu Tang) A.H.Wang & L.Tan.

## Introduction

The genus *Fagopyrum* Miller (Polygonaceae) includes more than 20 species (Tian et al. 2011; Min et al. 2023; Li et al. 2023) with southwest China serving as its center of distribution and diversity (Tang et al. 2019; Fan et al. 2021). Except for *F. tataricum* (L.) Gaertn. and *F. esculentum* Moench, all *Fagopyrum* species are wild (Ohsako and Li 2020; Zhou et al. 2021). Species identification within *Fagopyrum* has traditionally relied on classical morphological characters (e.g., leaves simple, alternate; leaf blade triangular, cordate, broadly ovate, sagittate, or linear; inflorescence racemose or corymbose; flowers bisexual; perianth persistent, 5-parted; achenes trigonous, not winged or horned at base.) (Li et al. 2003; Ohsako and Li 2020; Zhou et al. 2021). However, numerous studies have shown that defining the interspecific or intraspecific relationships within the genus *Fagopyrum* solely based on morphological characteristics is unreliable (Chen 1999; Wang et al. 2017; Tang et al. 2019; Min et al. 2023).

Apart from plant morphology, micromorphological characteristics can assist taxonomists in defining species boundaries in plants (Youngken 1949; Tomoshevich et al. 2022). For instance, pollen morphology is a crucial trait for plant taxonomic identification (Sampson 2000; Tomoshevich et al. 2022), with characteristics such as shape, size, pore structure, symmetry, and ornamentation being highly conserved and commonly used to resolve taxonomic challenges (Sampson 2000; Mander and Punyasena 2018). Compared with external morphological traits, anatomical structures exhibit greater genetic stability and thus hold irreplaceable value in plant classification (Chatelet et al. 2013; Sinnott-Armstrong et al. 2022). Therefore, pollen and anatomical characteristics can provide valuable insights for elucidating interspecific relationships within *Fagopyrum* species.

Molecular phylogenetic reconstruction based on DNA sequences is an effective approach for clarifying the interspecific relationships among plants (Sun et al. 2008; Hu et al. 2015; Rajput et al. 2025). Internal transcribed spacer (ITS) sequences have been widely employed to investigate phylogenetic relationships at lower taxonomic levels (Oxelman 1996; Jafari et al. 2022). In angiosperms, chloroplast DNA (cpDNA) is predominantly maternally inherited, evolves at a relative slow rate due to its highly conserved genome, and does not undergo recombination in higher plants (Olsson et al. 2018; Huynh et al. 2021). Therefore, the combination of multiple genes in phylogenetic analyses is often favored to enhance the accuracy and robustness of phylogenetic inference (Liao et al. 2014; Kadam et al. 2023).

*Fagopyrum caudatum* var. grandiflorum M.L.Zhou & Yu Tang was described in 2023 from Longnan City, Gansu Province, China. It was treated as a variety of *F. caudatum* (Li et al. 2023) primarily because of its larger flowers. However, our field investigations revealed that, beyond flower size, multiple additional morphological differences exist between the two taxa. In this study, we aim to clarify the taxonomic status of *F. caudatum* var. grandiflorum by integrating morphological, palynological, anatomical, and molecular evidence.

## Materials and methods

### Plant materials

For this study, leaves and seeds of *F. caudatum* and *F. caudatum* var. grandiflorum were collected from Sichuan and Gansu Province, China (see sample information in Suppl. material 1: table SS1). Voucher specimens of both taxa were deposited in the Herbarium of Xichang University (XIAS), Xichang University, China. Leaf samples were dried and preserved in silica gel. After the seeds are dried, they are preserved in 4 °C until further use. Chromosomes were performed using drop methods using root tips in the mitosis metaphase (Chen 2001).

### Scanning electron microscope of pollen and paraffin section of leaf

The seeds of *F. caudatum* and *F. caudatum* var. grandiflorum were planted under the identical conditions of light, moisture, and nutrients. During the flowering period, mature anthers were collected, fixed in 2.5% glutaraldehyde, and stored at 4 °C. Simultaneously, the fifth or sixth leaf from the top was sampled and fixed in 70% formaldehyde-acetic acid-ethanol fixative solution. Subsequently, pollen electron microscope scanning and leaf paraffin section were carried out by Keruinuo Biotechnology Co., Ltd. (Wuhan, China). Thereafter, pollen morphology and leaf anatomical structure parameters were measured using IMAGE J. For each taxon, 30 pollen grains and leaves of three individual plants were analyzed. Finally, one-way ANOVA was performed using ORIGIN 2021. Pollen shape and size was classified according to Erdtman (1952), murus width and lumina density are based on Mao et al. (2024).

### Sequence amplification and phylogenetic analyses

For molecular analyses, total genomic DNA was extracted from dried leaves using the Plant Genome DNA Kit (TIANGEN, Beijing, China). We then amplified three DNA regions using available primers: *matK* (Zheng 2012), *psbA*-*trnH* (Aldrich et al. 1988), and ITS (White et al. 1990). The primers and PCR conditions for the ITS, *matK*, and *psbA*-*trnH* sequencing are listed in Table 1. Purified products were sequenced by Sangon Biological Engineering and Technology Service Ltd. (Shanghai, China). In addition, we included ITS, *matK*, and *psbA*-*trnH* sequences of the remaining species in *Fagopyrum* and related genera from GenBank (see Suppl. material 1: table SS2).

**Table 1.** The primers and PCR conditions used in this study.

Molecular marker	Primer	Sequence (5'-3')	PCR conditions
*ITS*	ITS4	TCCTCCGCTTATTGATATGC	1 cycle: 95 °C 4 min; 35 cycles: 94 °C 30 s, 51 °C 45 s, 72 °C 1 min; 1 cycle: 72 °C 8 min.
	ITS5	TCCGTAGGTGAACCTGCGG	
*matK*	F	ATGGAGGAATTCCAAGGATATTTA	1 cycle: 95 °C 4 min; 35 cycles: 94 °C 40 s, 56 °C 45 s, 72 °C 1 min; 1 cycle: 72 °C 10 min.
	R	TCAATCATTATGACTGGCCAAA	
*psbA*-*trnH*	F	CGCGCATGGTGGATTCACAATCC	1 cycle: 95 °C 4 min; 35 cycles: 94 °C 1 min, 59 °C 1 min, 72 °C 1 min; 1 cycle: 72 °C 8 min.
	R	GTTATGCATGAACGTAATGCTC	

DNA sequences were first checked using BLAST nucleotide alignment on NCBI database. Subsequently, multiple sequence alignments were performed using MAFFT (Katoh and Standley 2013). We created two separate alignments: a nuclear dataset of the ITS region, and a concatenated plastid matrix including *matK*, and *psbA*-*trnH* sequences. The concatenation of the plastid sequences was performed with PHYML 3.1 (Guindon et al. 2009). Appropriate DNA substitution models and gamma rate heterogeneity were determined using JMODETEST v3.06 (Posada and Crandall 1998) based on the Akaike Information Standard (AIC). The best-fitting models were TIM3 + G for ITS, and TVM + G for the combined matrices (*matk* + *psbA*-*trnH*). Phylogenetic analyses were then conducted using the maximum-likelihood (ML) approach implemented in PHYML 3., with statistical support for nodes estimated from 1,000 fast bootstrap replicates. In addition, Bayesian inference was performed using MRBAYES 3.1.2 (Huelsenbeck and Ronquist 2001), applying the same evolutionary models as those used in the ML analyses.

## Results

### Morphological traits measurements

**Figure 1.**
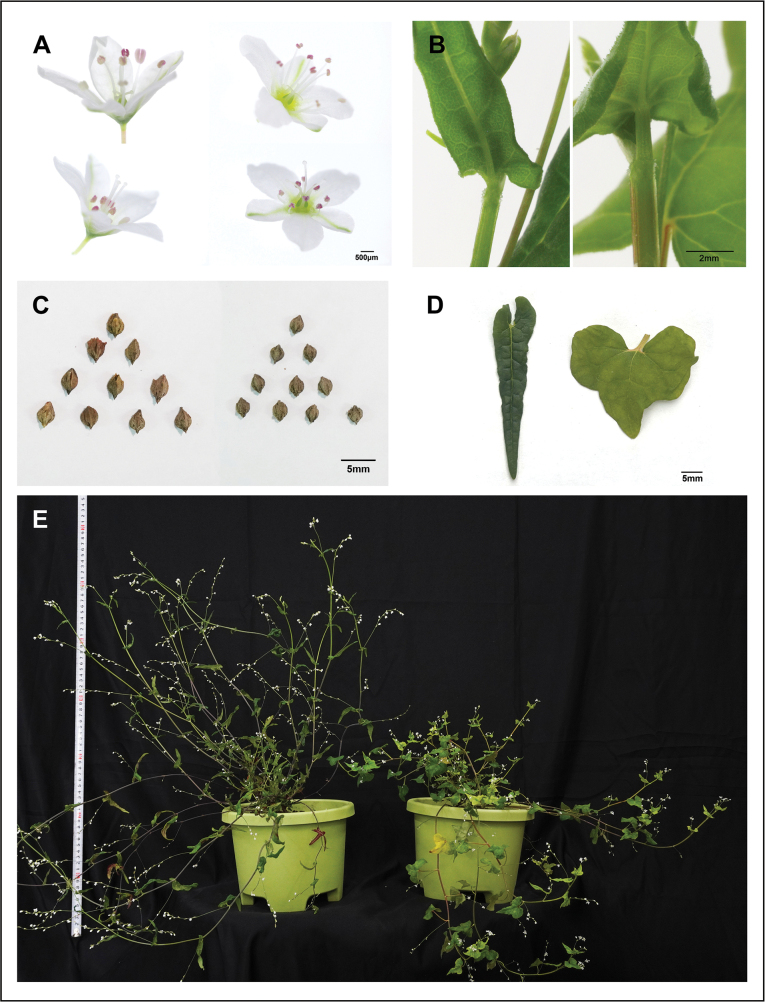
Morphological characteristics of *F. caudatum* var. grandiflorum (left) and *F. caudatum* (right). **A**. Flower; **B**. Petiole; **C**. Achene; **D**. Leaf; **E**. Whole plant.

Morphological measurements were conducted to assess phenotypic differentiation between *F. caudatum* and *F. caudatum* var. grandiflorum. Overall, the two taxa differ in floral, achene, and leaf traits (Fig. 1A–D). Both taxa exhibit heterostyly (i.e., a stylar polymorphism in which a species or population contains floral morphs with reciprocal style and stamens heights; Fig. 1A), although their floral morphology differs. The mean floral diameter of *F. caudatum* is 5.23 ± 0.43 mm, slightly smaller than that of *F. caudatum* var. grandiflorum (5.43 ± 0.42 mm). In *F. caudatum*, petiole length decreases progressively toward the distal end of the branch, whereas in *F. caudatum* var. grandiflorum, petioles gradually shorten until absent (Fig. 1B). Achene measurements further revealed that *F. caudatum* var. grandiflorum (length: 3.90 ± 4.03 mm, width: 2.64 ± 3.20 mm) produces significantly larger than *F. caudatum* (length: 3.51 ± 3.02 mm, width: 2.08 ± 1.74 mm; p < 0.05) (Fig. 1C). Additionally, the leaves of *F. caudatum* are triangular-hastate, whereas those of *F. caudatum* var. grandiflorum are narrowly hastate (Fig. 1D). Finally, observations of root tip cells at mitotic metaphase revealed that both *F. caudatum* and *F. caudatum* var. grandiflorum contain 16 chromosomes, with 2*n* = 2*x* = 16 (Suppl. material 2: fig. S1).

### Pollen morphology

**Figure 2.**
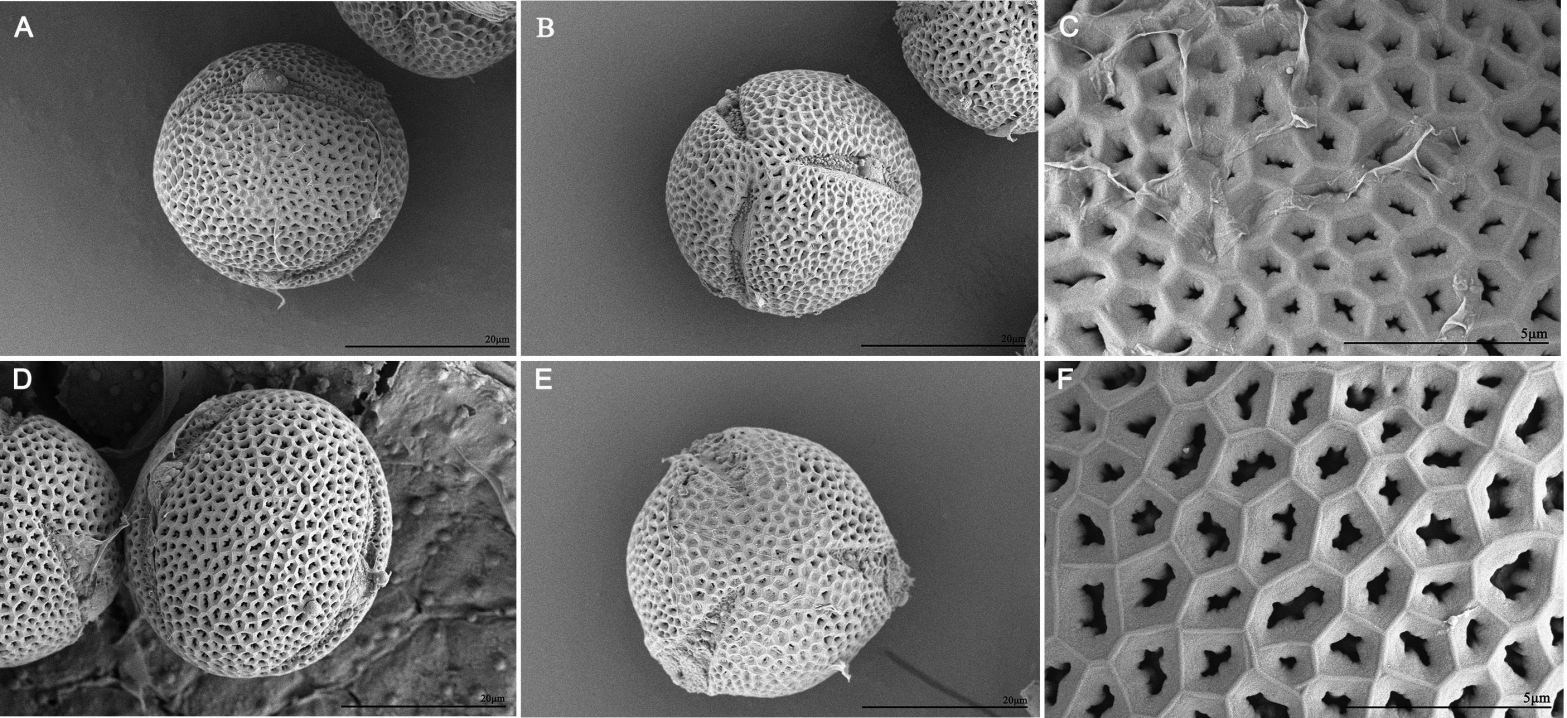
Electron microscopic results of pollen from *F. caudatum* var. grandiflorum and *F. caudatum*. **A–C**. *F. caudatum* var. grandiflorum; **D–F**. *F. caudatum*. **A, D**. Equatorial view; **B, E**. Polar view; **C, F**. Exine sculpture. Scale bars are indicated in the lower right corner of each image.

The pollen grains of *F. caudatum* and *F. caudatum* var. grandiflorum are monad, spheroidal, and 3-zonocolporate (NPC-345), with reticulate exine ornamentation (Fig. 2, Suppl. material 1: table S3). Although pollen grains of both taxa fall within the medium size range, those of *F. caudatum* (polar axis: 30.84 ± 1.71 µm, equatorial axis: 31.94 ± 1.55 µm) are larger than those of *F. caudatum* var. grandiflorum (polar axis: 29.71 ± 1.32 µm, equatorial axis: 30.8 ± 1.68 µm) (p < 0.05) (Fig. 2, Suppl. material 1: table S3). Furthermore, the pollen apertures of *F. caudatum* (length: 27.52 ± 1.4 µm, width: 3.87 ± 0.68 µm) are longer but narrower than those of *F. caudatum* var. grandiflorum (length: 25.95 ± 2.13 µm, width: 4.44 ± 0.70 µm) (Fig. 2, Suppl. material 1: table S3). The lumina are irregular in both taxa (Fig. 2). There was little difference in the width of murus; however, the lumina number of per 10 μm^2^ in *F. caudatum* var. grandiflorum (65.75 ± 1.27 /10 μm^2^) was significantly higher than that in *F. caudatum* (48.90 ± 2.19 /10 μm^2^) (Suppl. material 1: table S3). Those indicate that the lumina of *F. caudatum* var. grandiflorum is smaller than that of *F. caudatum*.

### Leaf anatomical structures

Leaf anatomical structures differ significantly between *F. caudatum* and *F. caudatum* var. grandiflorum (Table 2, Suppl. material 2: fig. S2). The thickness of the upper epidermis in *F. caudatum* var. grandiflorum (27.55 ± 6.21 µm) is significantly lower than that in *F. caudatum* (39.27 ± 7.74 µm; p < 0.05) (Table 2). A similar pattern is found in the lower epidermis thickness, with *F. caudatum* var. grandiflorum (19.07 ± 3.95 µm) exhibiting significantly reduced thickness compared with *F. caudatum* (24.30 ± 4.49 µm; p < 0.05) (Table 2). The palisade tissue in *F. caudatum* var. grandiflorum (70.45 ± 9.10 µm) is also significantly thinner than in *F. caudatum* (99.01 ± 20.77 µm; p < 0.05) (Table 2), as is the spongy tissue (46.54 ± 6.77 µm vs 65.68 ± 13.16 µm; p < 0.05) (Table 2). In contrast, overall leaf thickness is significantly greater in *F. caudatum* (216.58 ± 33.74 µm) than in *F. caudatum* var. grandiflorum (171.59 ± 17.29 µm) (p < 0.05). However, the ratio of palisade tissue to spongy tissue does not differ significantly between the two taxa.

**Table 2.** Leaf anatomical traits of *F. caudatum* var. grandiflorum and *F. caudatum*.

Taxa	Thickness of upper epidermis (μm)	Thickness of lower epidermis (μm)	Leaf thickness (μm)	Thickness of palisade tissue (μm)	Thickness of spongy tissue (μm)	Palisade tissue and spongy tissue ratio	Thickness of midrib (μm)
*F. caudatum* var. grandiflorum	27.55 ± 6.21b	19.07 ± 3.95b	171.59 ± 17.29b	70.45 ± 9.10b	46.54 ± 6.77b	1.54 ± 0.27a	461.49 ± 18.57a
*F. caudatum*	39.27 ± 7.74a	24.30 ± 4.49a	216.58 ± 33.74a	99.01 ± 20.77a	65.68 ± 13.16a	1.54 ± 0.35a	434.15 ± 82.21a

Note: Data are presented as mean ± standard deviation. Different lowercase letters within the same column indicate significant differences among treatments at the 0.05 level.

### Phylogenetic analyses: nuclear and plastid phylogenetic reconstructions

**Figure 3.**
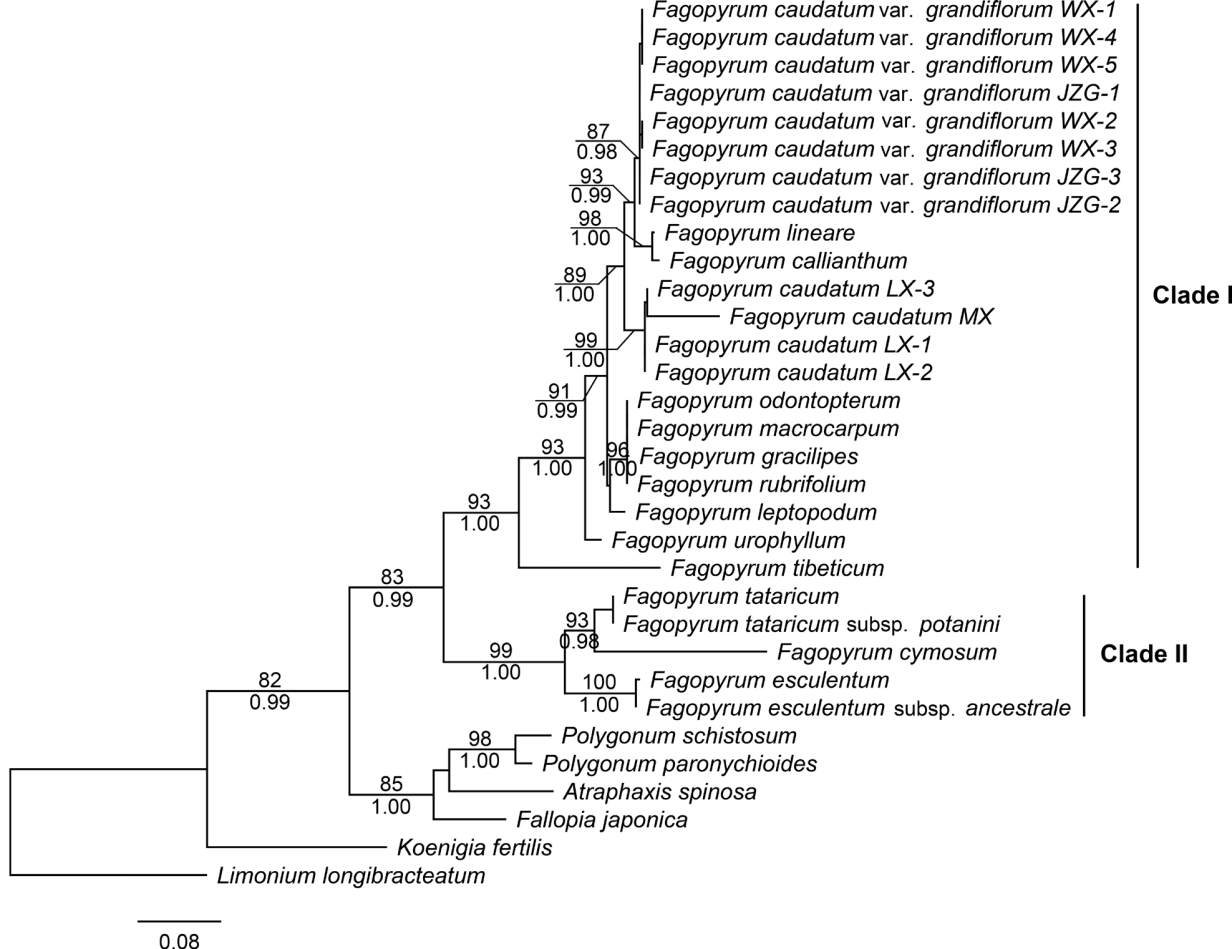
Maximum likelihood tree inferred from ITS sequences. Bootstrap support (BS > 50%) and Bayesian posterior probabilities (PP > 90%) are indicated above and below the branches, respectively. Capital letters following species names denote the origin locations, and numbers represent individual samples.

The nuclear ITS dataset comprised 32 sequences for phylogenetic analysis, with *Limonium longibracteatum* Erben designated as the outgroup. Both ML and BI analyses, recovered *Fagopyrum* as a monophyletic genus, with all species forming a single well-supported clade (BS = 83%, PP = 0.99) (Fig. 3). Within *Fagopyrum*, the nuclear phylogeny resolved two strongly supported subclades, designated Clade I (BS = 93%, PP = 1.00) and Clade II (BS = 99%, PP = 1.00), respectively. Within Clade I, all *F. caudatum* accessions form a monophyletic group (BS = 99%, PP = 1.00), whereas *F. caudatum* var. grandiflorum clusters with *F. lineare* (Sam.) Haraldson and *F. callianthum* Ohnishi (BS = 93%, PP = 0.99) (Fig. 3). Moreover, accessions of *F. caudatum* var. grandiflorum form a well-supported subclade within this cluster (BS = 87%, PP = 0.98) (Fig. 3).

**Figure 4.**
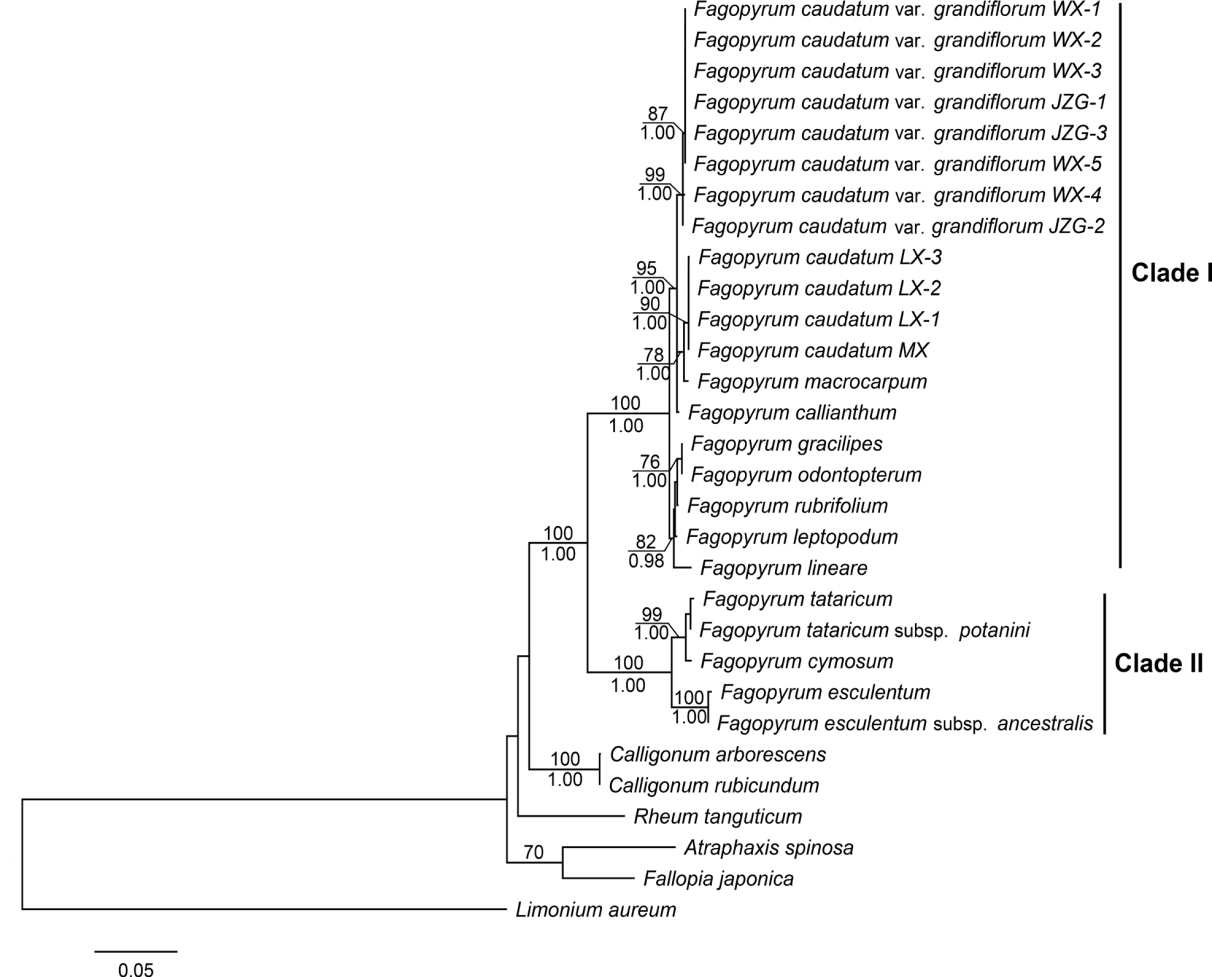
Maximum likelihood tree inferred from plastid sequences (*matK* + *pabA*-*trnH*). Bootstrap support (BS > 50%) and Bayesian posterior probabilities (PP > 90%) are indicated above and below the branches, respectively. Capital letters following species names denote the origin locations, and numbers represent individual samples.

The plastid dataset comprised 30 sequences for phylogenetic analysis, with *Limonium aureum* (L.) Hill ex Kuntze as the outgroup. Whether inferred from individual loci or a combined dataset, all *Fagopyrum* species form a single, well-supported clade (BS = 100%, PP = 1.00) (Fig. 4). Within this clade, *Fagopyrum* species are further divided into two strongly supported subclades, designated Clade I (BS = 100%, PP = 1.00) and Clade II (BS = 100%, PP = 1.00) (Fig. 4). Within Clade I, sequences of *F. caudatum* cluster with *F. macrocarpum* Ohsako & Ohnishi, 1998(BS = 78%, PP = 1.00), whereas *F. caudatum* var. grandiflorum forms a strongly supported subclade (BS = 99%, PP = 1.00) (Fig. 4).

## Discussion

### Differences in morphological characteristics

*Fagopyrum caudatum* is a perennial herbaceous plant primarily distributed in Yunnan, Sichuan, and Gansu Provinces of China (Li et al. 2003; Xia and Wang 2008). *Fagopyrum caudatum* var. grandiflorum, also a perennial herbaceous plant, was described from Longnan City, Gansu Province, China, in 2023 (Li et al. 2023). The two taxa mainly differ in the flowers’ size, with *F. caudatum* var. grandiflorum bearing larger flowers (Li et al. 2023). In the present study, in addition to Longnan City, *F. caudatum* var. grandiflorum from Jiuzhaigou (Sichuan Province, China) was also collected. When cultivated under identical conditions, several morphological differences between the two taxa were observed. First, although the flowers of *F. caudatum* var. grandiflorum were larger, the difference was not statistically significant. In contrast, the achenes of *F. caudatum* var. grandiflorum were significantly larger. Leaf morphology also differed notably: leaves of *F. caudatum* var. grandiflorum are narrowly and elongated hastate, whereas those of *F. caudatum* are triangular-hastate. Towards the branch apex, petioles of *F. caudatum* var. grandiflorum became gradually shorter until absent, whereas in *F. caudatum* they also shorten but remain present. Cytological observation results of root tip further revealed that both taxa are diploid (2*n* = 2*x* = 16). Collectively, differences in flower and achene size, leaf morphology, and petiole characteristics clearly distinguish *F. caudatum* var. grandiflorum from *F. caudatum*.

### Differences in pollen morphology

Pollen morphological characteristics vary among species of *Fagopyrum* (Zhou et al. 2003; Hu et al. 2025). In the present study, pollen grains of *F. caudatum* and *F. caudatum* var. grandiflorum share diagnostic features typical of the genus, including a spheroidal shape, 3-zonocolporate apertures, and reticulate exine ornamentation (Zhou et al. 2003; Hu et al. 2025). However, pollen grains of *F. caudatum* var. grandiflorum are significantly smaller than those of *F. caudatum*. Although both taxa exhibit reticulate exine ornamentation, the lumina are significantly smaller in *F. caudatum* var. grandiflorum than in *F. caudatum*.

### Differences in leaf anatomical structure

Leaf anatomical traits are generally considered genetically stable and thus provide valuable characters for plant classification (Youngken 1949; Sinnott-Armstrong et al. 2022). In this study, the upper epidermis, lower epidermis, palisade tissue, spongy tissue, and leaf thickness of *F. caudatum* are greater than those of *F. caudatum* var. grandiflorum. Leaf anatomical structure is closely associated with environmental adaptability, such as drought, cold, and high stress (Rindyastuti et al. 2018; Zhang et al. 2024). These differences in leaf structure suggest that *F. caudatum* possess stronger adaptability and stress resistance compared to *F. caudatum* var. grandiflorum, which could partly explain its broader geographic distribution.

### Phylogenetic relationships

The *Fagopyrum* species can be divided into two groups, namely Cymosum subgroup and Urophyllum subgroup (Chen 1999; Hu et al. 2016). Our research results also support this view, and both *F. caudatum* var. grandiflorum and *F. caudatum* belong to the Urophyllum subgroup. Previous chloroplast-based studies indicated a close relationship between *F. caudatum* hand *F. callianthum* Ohnishi (Yang 2008). In the present study, the phylogenetic analysis based on ITS sequences shows that both *F. caudatum* and *F. caudatum* var. grandiflorum are closely related to *F. callianthum* and *F. lineare*, with the latter two taxa more closely associated with *F. caudatum* var. grandiflorum. In contrast, plastid phylogenies place *F. caudatum* and *F. caudatum* var. grandiflorum in close proximity to *F. macrocarpum* and *F. callianthum*, with *F. caudatum* showing a particularly close affinity to *F. macrocarpum*. However, both the ITS and the plastid datasets clearly distinguish *F. caudatum* and *F. caudatum* var. grandiflorum across different geographical origins, indicating substantial genetic divergence. These results support the interpretation that their relationship is interspecific rather than intraspecific.

Furthermore, *F. caudatum* var. grandiflorum is morphologically and geographically distinct from *F. lineare* and *F. callianthum* (Li et al. 2003; Zhou et al. 2021). *Fagopyrum lineare* has slender stems and linear leaves, and is restricted to Dali Bai Autonomous Prefecture of Yunnan Province (China) (Li et al. 2003; Zhou et al. 2021). The leaves of the *F. callianthum* are heart-shaped with grayish spots, and have only been found in Aba Tibetan and Qiang Autonomous Prefecture of Sichuan Province (Zhou et al. 2021). Therefore, *F. caudatum* var. grandiflorum should be treated as an independent species.

### Classification of *Fagopyrum caudatum* var. grandiflorum

The results of morphological, palynological, anatomical, and phylogenetic analysis all indicate that *F. caudatum* var. grandiflorum and *F. caudatum* are two distinct taxa. Therefore, we propose that *F. caudatum* var. grandiflorum should not be regarded as a variant of *F. caudatum*, but rather as an independent species. Classify as follows:

#### Fagopyrum grandiflorum

Taxon classificationPlantaeCaryophyllalesPolygonaceae

(M.L.Zhou & Yu Tang) A.H.Wang & L.Tan comb. et stat. nov.

7A54024A-42B1-53EA-AA35-EC2AAB654ED0

urn:lsid:ipni.org:names:77381821-1

Fagopyrum caudatum var. grandiflorum M.L.Zhou & Yu Tang, Phytotaxa 587(2): 201. 2023 (Li et al. 2023).

##### Holotype.

China. • Gansu Province, Wen County; 1052–1220 m; 18 Oct 2020; Zhou &Tang, 202012, CAAS2205!. ***Topotype***: China. • Gansu Province, Wen County; 1115 m; 9 Oct 2023; A.H.Wang & L.Tan, XIAS202307003!.

##### Description.

Herbs annual, 60–90 cm tall, branched at base or lower-middle, branches decumbent or ascending; Stems cylindrical, striate, green, internodes 1.5–8 cm; Single leaves alternate, leaves papery, narrowly hastate, 1.3–6 cm long, 0.4–3 cm wide, sparsely short hairy above, glabrous below, midvein slightly prominent abaxially; Petiole 0–1.2 cm, petioles gradually shorten until absent, green, glabrous; Ocrea membranous, 4–7 mm long, oblique, apex acute; Inflorescence terminal or axillary, racemose, very lax, 4–13 cm, several racemes aggregated and panicle-like; Bracts 2–3 mm, membranous, oblique, apex acuminate, each 2– or 3-flowered; Pedicel 2–4 mm, slender, glabrous, apex articulate; Perianth white, tepals elliptic or ovoid elliptic, 2.5–3.5 mm long, 1.5–2.0 mm wide, apex rounded, accrescent; Stamens 8, arranged in 2 rounds, outer round 5, inner round 3, anthers pink or red, elliptic; Ovary ovoid triangular, style 3; Achenes wrapped or slightly beyond in the persistent perianth, dark brown or black, oval or broadly ovoid, trigonous, 3.0–4.7 mm long, 2.0–3.1 mm in diameter (Suppl. material 2: figs S3, S4).

##### Habitat and distribution.

The plant grows in the valleys and mountain slopes of rocks and weeds at elevation 1000–1500 m and occurs in Wenxian County of Gansu Province and Jiuzhaigou County of Sichuan Province, China.

## Conclusions

*Fagopyrum caudatum* var. grandiflorum and *F. caudatum* have significant differences in morphological characteristics, pollen morphology, leaf anatomical structure, and phylogenetic relationships. Therefore, *F. caudatum* var. grandiflorum should not be treated as a variant of *F. caudatum*, but rather as an independent species and classified as *Fagopyrum grandiflorum*.

## Supplementary Material

XML Treatment for Fagopyrum grandiflorum

## Additional information

### Artificial Intelligence (AI) use

The authors accept full responsibility for the content of the manuscript, including the disclosure of any use of AI.

No AI tools were used in the preparation of this manuscript.

### Author ORCIDs

L. Tan https://orcid.org/0000-0002-4235-5382

H.-M. Du https://orcid.org/0000-0003-0183-5192

A. Valdés-Florido https://orcid.org/0000-0002-0325-0414

H. Chen https://orcid.org/0000-0002-7745-9069

### Data availability

All of the data that support the findings of this study are available in the main text or Supplementary Information.

## Supplementary materials

Supplementary material 1 Supplementary tables Data type docx Explanation note **table SS1**. Plant material used in this study. **table SS2**. Sequences used in phylogenetic analyses. **table S3**. Quantitative data of pollen grains of *Fagopyrum caudatum* var. grandiflorum and *F. caudatum*, n=30. https://binary.pensoft.net/file/1686535This dataset is made available under the Open Database License (http://opendatacommons.org/licenses/odbl/1.0/). The Open Database License (ODbL) is a license agreement intended to allow users to freely share, modify, and use this Dataset while maintaining this same freedom for others, provided that the original source and author(s) are credited. Lu Tan, Rong Yan, Han-Mei Du, Ying Peng, Zi-Xin Fan, Qing-Hai Wang, Ana Valdés-Florido, Hong Chen, Sheng-Chun Li, An-Hu Wang

Supplementary material 2 Supplementary figures Data type docx Explanation note **fig. S1**. Chromosome number of *F. caudatum* var. grandiflorum and *F. caudatum*. **fig. S2**. Leaf anatomical structures of *F. caudatum* var. grandiflorum. **fig. S3**. Specimen of *F. caudatum* var. grandiflorum. **fig. S4**. The tissue characteristics of *F. caudatum* var. grandiflorum. https://binary.pensoft.net/file/1686536This dataset is made available under the Open Database License (http://opendatacommons.org/licenses/odbl/1.0/). The Open Database License (ODbL) is a license agreement intended to allow users to freely share, modify, and use this Dataset while maintaining this same freedom for others, provided that the original source and author(s) are credited. Lu Tan, Rong Yan, Han-Mei Du, Ying Peng, Zi-Xin Fan, Qing-Hai Wang, Ana Valdés-Florido, Hong Chen, Sheng-Chun Li, An-Hu Wang
