# Supplementary material for: Revisiting the taxonomy of Fagopyrum caudatum var. grandiflorum (Polygonaceae) using morphological and molecular data
Source: PhytoKeys. 2026 Jun 18;276:261–74. doi: 10.3897/phytokeys.276.196051 (PMC13306200; doi:10.3897/phytokeys.276.196051)
Supplement: Supplementary material 1 — Supplementary tables [file phytokeys-276-261_article-196051__-s001.docx]

**Table S1.** Plant material used in this study.

| No. | Species | Origin | Origin Abbreviation |
| --- | --- | --- | --- |
| 1 | *Fagopyrum caudatum* | Maoxian, Sichuan, China | MX |
| 2 | *F. caudatum* | Lixian, Sichuan, China | LX |
| 3 | *F. caudatum* var. *grandiflorum* | Jiuzhaigou, Sichuan, China | JZG |
| 4 | *F. caudatum* var. *grandiflorum* | Wenxian, Gansu, China | WX |

**Table S2. Sequences used in phylogenetic analyses**

| **No.** | **Species** | **GenBank No.** | | |
| --- | --- | --- | --- | --- |
|  |  | **ITS** | **matK** | **psbA-trnH** |
| 1 | *Fagopyrum caudatum* (Sam.) A.J.Li (MX) | PX959869 | PX965557 | PX965585 |
| 2 | *F. caudatum* (Sam.) A.J.Li (LX-1) | PX959870 | PX965558 | PX965586 |
| 3 | *F. caudatum* (Sam.) A.J.Li (LX-2) | PX959871 | PX965559 | PX965587 |
| 4 | *F. caudatum* (Sam.) A.J.Li (LX-3) | PX959872 | PX965560 | PX965588 |
| 5 | *F. caudatum* var. *grandiflorum* M.L.Zhou & Yu Tang (JZG-1) | PX959873 | PX965561 | PX965589 |
| 6 | *F. caudatum* var. *grandiflorum* M.L.Zhou & Yu Tang (JZG-2) | PX959874 | PX965562 | PX965590 |
| 7 | *F. caudatum* var. *grandiflorum* M.L.Zhou & Yu Tang (JZG-3) | PX959875 | PX965563 | PX965591 |
| 8 | *F. caudatum* var. *grandiflorum* M.L.Zhou & Yu Tang (WX-1) | PX959876 | PX965564 | PX965592 |
| 9 | *F. caudatum* var. *grandiflorum* M.L.Zhou & Yu Tang (WX-2) | PX959877 | PX965565 | PX965593 |
| 10 | *F. caudatum* var. *grandiflorum* M.L.Zhou & Yu Tang (WX-3) | PX959878 | PX965566 | PX965594 |
| 11 | *F. caudatum* var. *grandiflorum* M.L.Zhou & Yu Tang (WX-4) | PX959879 | PX965567 | PX965595 |
| 12 | *F. caudatum* var. *grandiflorum* M.L.Zhou & Yu Tang (WX-5) | PX959880 | PX965568 | PX965596 |
| 13 | *F. cymosum* (Trevir.) Meisn. | DQ780602* | JF829983* | JQ807571* |
| 14 | *F. esculentum* Moench | PX352073 | JF829981* | EF653736* |
| 15 | *F. esculentum* subsp. *ancestralis* Ohnishi WX | PX352069 | PX400587 | PX412691 |
| 16 | *F. gracilipes* (Hemsl.) Dammer | JF829988* | PX400585 | JQ807568* |
| 17 | *F. odontopterum* Gross | MT672487* | PX400574 | JQ807566* |
| 18 | *F. leptopodum* (Diels) Hedberg | PX352076 | PX400575 | KY206925* |
| 19 | *F. macrocarpum* Ohsako & Ohnishi | PX352074 | PX400583 | MT668931* |
| 20 | *F. rubrifolium* Ohsako & Ohnishi | MT644600* | PX400582 | PX412695 |
| 21 | *F. tataricum* (L.) Gaertn. | DQ780601* | JF829984* | KY206928* |
| 22 | *F. tataricum* subsp. *potanini* | AB000340* | PX400578 | PX412692 |
| 23 | *F. lineare* (Sam.) Haraldson | JF829993* | PX400577 | KY206926* |
| 24 | *F. callianthum* Ohnishi | AB000322* | PX400586 | JQ807574* |
| 25 | *F. tibeticum* (A.J.Li) Adr.Sanchez & Jan.M.Burke | JN187103* | - | - |
| 26 | *Atraphaxis spinosa* Laxm | KJ707515* | EU840453* | PV898517* |
| 27 | *Calligonum arborescens* Litv | - | EU840494* | MN449333* |
| 28 | *C. rubicundum* Bunge | - | EU840493* | MZ303138* |
| 29 | *Fallopia japonica* (Houtt.) Ronse Decr | EU808015* | EU024772* | EU554048* |
| 30 | *Koenigia fertilis* Maxim | JQ360832* | - | - |
| 31 | *Limonium aureum* (L.) Hill ex Kuntze | - | MF190430* | PV898492* |
| 33 | *L. longibracteatum* Erben | MH582617* |  |  |
| 34 | *Polygonum schistosum* Czukav. | GQ340054* | - | - |
| 35 | *P. paronychioides* C.A.Mey. | GQ340029* | - | - |
| 36 | *Rheum tanguticum* Maxim. ex Balf | KF514623* | AB115683* | PP858648* |

**Note:** -, no data; *, data from published sequences from GenBank.

Table S3. Quantitative data of pollen grains of *Fagopyrum caudatum* var. *grandiflorum* and *F. caudatum*, n=30.

| **Taxa** | **Polar axis**  **(µm)** | **Equatorial diameter**  **(µm)** | **P/E** | **Pollen shape** | **Pollen size** | **Colpus length**  **(µm)** | **Colpus width**  **(µm)** | **Murus width**  **(µm)** | **Lumina density**  **(/10μm^2^)** | **Surface sculpturing** | **Aperature** |
| --- | --- | --- | --- | --- | --- | --- | --- | --- | --- | --- | --- |
| *F. caudatum* var. *grandiflorum* | 29.71±1.32b | 30.8±1.68b | 0.96±0.05a  (0.91-1.07) | Spheroidal | Medium | 25.95±2.13b | 4.44±0.70a | 0.87±0.12a | 65.75±1.27a | Reticulate | Tricolporate |
| *F. caudatum* | 30.84±1.71a | 31.94±1.55a | 0.97±0.07a  (0.74-1.11) | Spheroidal | Medium | 27.52±1.4a | 3.87±0.68b | 0.86±0.12a | 48.90±2.19b | Reticulate | Tricolporate |

Note: Data are presented as mean ± standard deviation. Different lowercase letters within the same column indicate significant differences among treatments, P < 0.05.
