# Supplementary material for: Revisiting the taxonomy of Fagopyrum caudatum var. grandiflorum (Polygonaceae) using morphological and molecular data
Source: PhytoKeys. 2026 Jun 18;276:261–74. doi: 10.3897/phytokeys.276.196051 (PMC13306200; doi:10.3897/phytokeys.276.196051)
Supplement: Supplementary material 2 — Supplementary figures [file phytokeys-276-261_article-196051__-s002.docx]

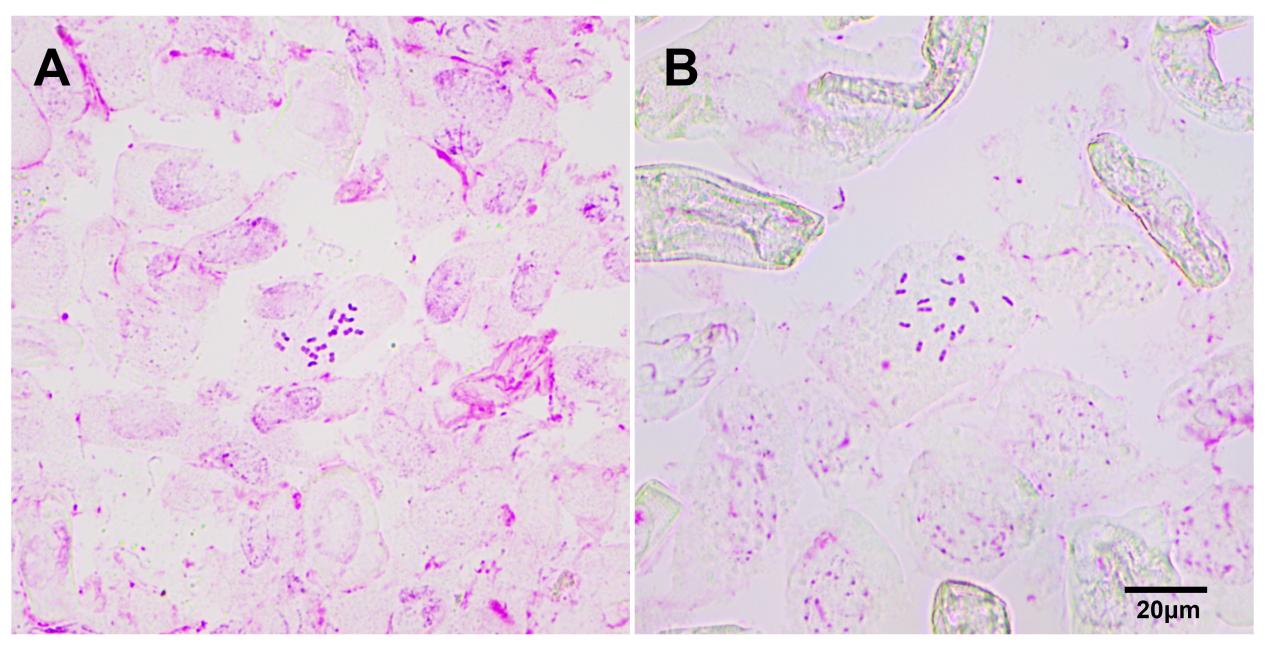


**Figure S1.** Chromosome number of *F. caudatum* var. *grandiflorum* and *F. caudatum*. A: *F. caudatum* var. *grandiflorum*; B: *F. caudatum*. Scale bar is indicated in the lower right corner of the image.


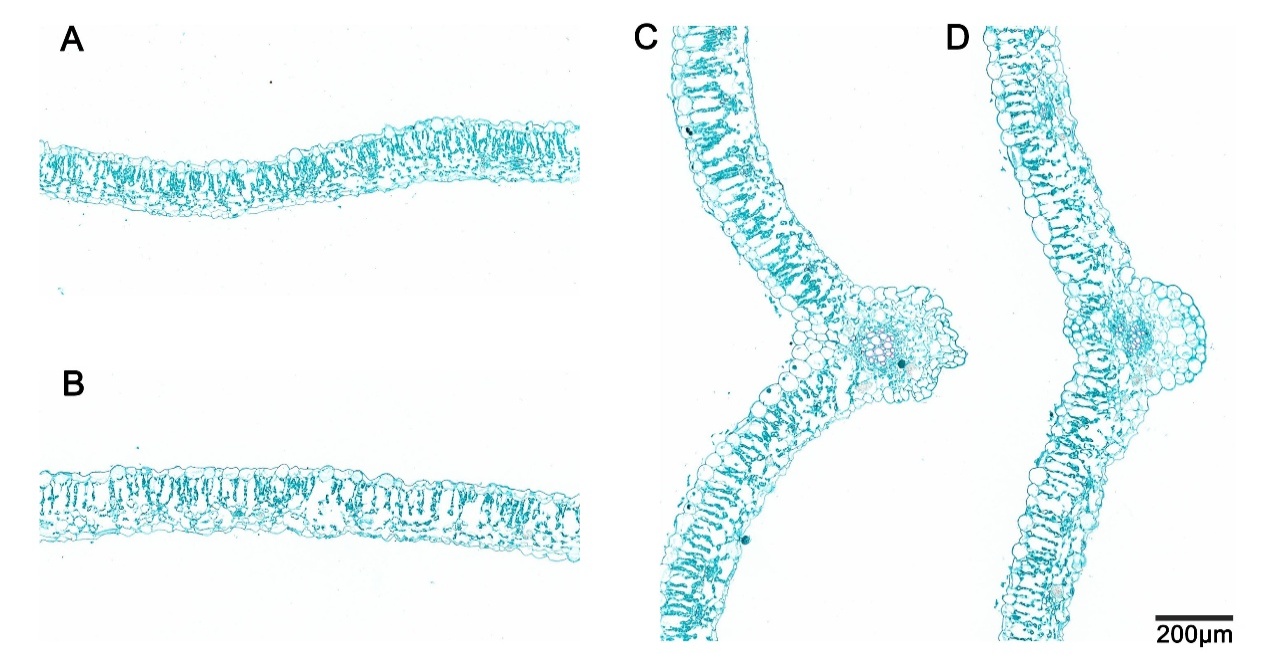


**Figure S2.** Leaf anatomical structures of *F. caudatum* var. *grandiflorum*. A, C: *F. caudatum* var. *grandiflorum*; B, D: *F. caudatum*. A, B: Transverse section of leaves; C, D: Transverse section of main vein of leaves. Scale bar is indicated in the lower right corner of the image.


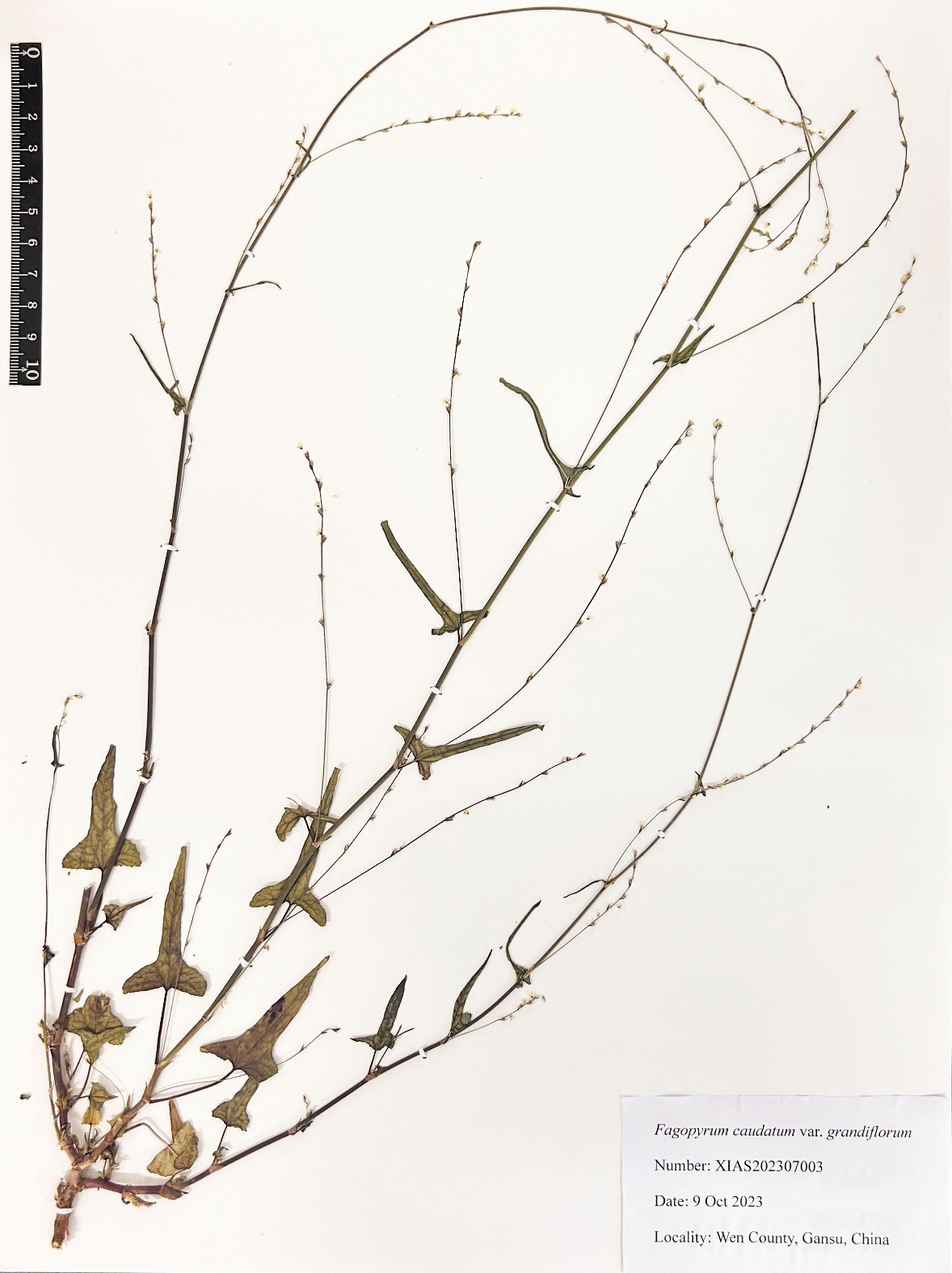


**Figure S3.** Specimen of *F. caudatum* var. *grandiflorum*.


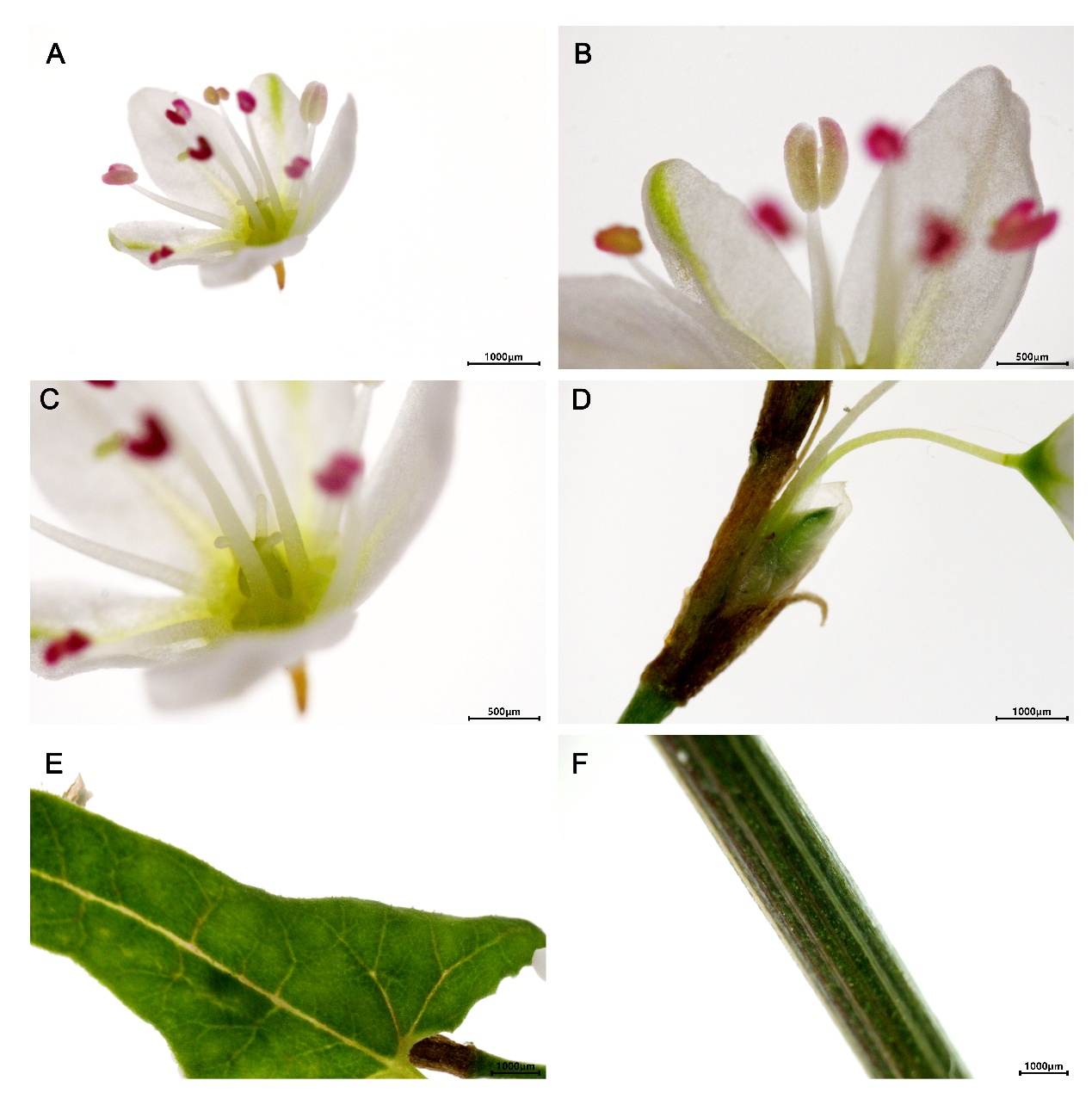


**Figure S4.** The tissue characteristics of *F. caudatum* var. *grandiflorum*. A: stamen; B: anther; C: style; D: bract and pedicel; E: upper surface of the leaf; F: stem. Scale bar is indicated in the lower right corner of the image.
